# A comparison of SARS-CoV-2 RNA extraction with the QuickGene-810 Nucleic Acid Isolation System compared to the EZ1 Advanced DSP Virus Kit

**DOI:** 10.1099/acmi.0.000353

**Published:** 2022-05-27

**Authors:** Thuy Phan, Rebecca Stephenson, Tina Cai, Nikol Andacic, Genevieve McKew

**Affiliations:** ^1^​ NSW Health Pathology, Department of Microbiology and Infectious Diseases, Concord Repatriation and General Hospital, Hospital Rd, Concord, NSW 2139, Australia; ^2^​ Concord Clinical School, Faculty of Medicine and Health, The University of Sydney, Sydney, Australia

**Keywords:** SARS-CoV-2, RNA extraction, QuickGene-810

## Abstract

The QuickGene-810 Nucleic Acid Isolation System is a semi-automated extraction platform which may be used for RNA extraction. New methods were required to support the rapid increase in respiratory virus testing during the SARS-CoV-2 pandemic. The aim of this study was to assess SARS-CoV-2 RNA extraction using the QuickGene-810 kit compared to the EZ1 Advanced Extraction Platform for use on the AusDiagnostics SARS-CoV-2, Influenza and RSV 8-well RT-PCR assay. Qualitative results from all clinical samples were concordant between the QuickGene-810 and the EZ1 extraction methods, demonstrating that the QuickGene-810 kit is suitable for use in pathogen diagnostics. However, there was an average difference of approximately two cycles between the cycle threshold (Ct) values for both SARS-CoV-2 targets, suggesting that the EZ1 kit yields a higher concentration of nucleic acid extract, possibly related to its use of carrier RNA and/or smaller elution volume, which infers the possibility of false negative results for samples with very low viral loads.

## Data Summary

The supporting data for this article can be found here on Figshare: https://doi.org/10.6084/m9.figshare.19316048 [1].

## Introduction

The COVID-19 pandemic, due to the worldwide spread of SARS-CoV-2, has led to unprecedented numbers of diagnostic molecular tests carried out in clinical laboratories [2]. High-throughput molecular diagnostic testing is an essential component of the public health response to the pandemic, facilitating healthcare facility preparedness, and population viral surveillance and control [2]. In early 2020, the rapid upscaling of molecular testing, mostly via laboratory-developed or commercial RT-PCR assays, meant a shortage of PCR kits and reagents [3]. In our laboratory, new extraction platforms and reagents had to be sourced and validated in near real-time. The QuickGene-810 Nucleic Acid Isolation System (Kurabo) (‘QuickGene-810’) is a semi-automated extraction platform that was introduced for use as an additional RNA extraction method to support the increase in respiratory virus testing during the SARS-CoV-2 pandemic. Little has been published on this extraction platform in the arena of pathogen diagnostics.

The QuickGene-810 is a compact bench-top machine for the rapid isolation of DNA or RNA from various samples, including human tissue samples. Using the RNA Tissue Kit SII (Kurabo), it processes eight samples at once, taking 30 min from start (sample preparation) to finish (nucleic acid elution). This platform did not utilize carrier RNA as part of the extraction process, which may affect the recovery of RNA from clinical samples [4]. The EZ1 Advanced and Advanced XL Extraction Platforms (Qiagen) are automated bench-top instruments that perform purification and extraction of nucleic acids from a broad range of sample types, including human respiratory samples. The EZ1 DSP viral extraction kit (Qiagen) uses magnetic bead technology to extract both RNA and DNA, and the platforms process six or 14 (Advanced XL) samples in 40 min. The EZ1 kit also contains carrier RNA as a reagent additive, which acts to enhance binding of viral nucleic acids and to prevent RNA degradation.

The aim of this study was to compare the accuracy of SARS-CoV-2 RNA extraction using the QuickGene-810 platform compared to the EZ1 DSP viral extraction kit and the EZ1 Advanced (or XL) Extraction Platform for nucleic acid extraction (Qiagen) (‘EZ1’) when samples were tested on the AusDiagnostics SARS-CoV-2, Influenza and RSV 8-well assay (AusDiagnostics).

The QuickGene-810 system was selected for comparison due to the ability to procure this platform and testing kits, during the supply chain disruption that was being experienced worldwide. The reference EZ1 Advanced Extraction platform was already an established platform in the laboratory and in diagnostic microbiology, and testing kits were available for use at the time of this study. Similarly, the AusDiagnostics High-Plex 24 System was an established platform within the laboratory. Procurement of the AusDiagnostics SARS-CoV-2, Influenza and RSV 8-well assay was secured, and due to local manufacturing was minimally disrupted by international supply issues.

## Methods

### Study setting and study design

The study was conducted in Concord Repatriation General Hospital’s Microbiology Department in March 2020. A convenience sample of 110 SARS-CoV-2-positive and -negative clinical samples from four different laboratories serving major public hospital networks in Sydney, Australia, during the first wave of the COVID-19 outbreak from February to March 2020 was collected. Samples were retrospectively tested on the SARS-CoV-2, Influenza and RSV 8-well assay (‘the assay’) after viral RNA extraction with the QuickGene-810 and the EZ1 kits. Overall agreement, and positive and negative agreement were calculated to compare extraction efficiency. The difference between Ct values was also calculated. A limit of detection analysis was conducted on three samples.

### Specimen collection and storage

The sample types were predominantly respiratory swabs (nasal, throat or nasopharyngeal) collected in UTM viral transport medium (Copan Diagnostics). One expectorated sputum sample that had been treated with Remel Sputasol (0.54% DTT; ThermoFisher Scientific) was included. All sample types were deemed acceptable for use on the assay as per the instructions for use (IFU). Samples were stored at 4 °C prior to testing.

### Nucleic acid extraction

The reference extraction platform used for comparison was the Qiagen EZ1 Advanced Extraction Platform with the EZ1 DSP Virus Kit, which effectively extracts both DNA and RNA. Both these Qiagen products have been previously validated for *in vitro* use with the assay [5]. An aliquot of 200 µl of pooled or neat sample was extracted to elute volumes of 60 µl (EZ1) or 100 µl (QuickGene-810). The assay’s Extraction Control (AusDiagnostics) was included in all samples prior to extraction, and EZ1 Viral carrier RNA (Qiagen) in those extracted with the EZ1 kit.

### Multiplex tandem PCR and analysis

The SARS-CoV-2, Influenza and RSV-8-well assay qualitatively detects SARS-CoV-2 *orf*1a and *orf*8, influenza A haemagglutinin surface protein (H1, H3, H5 and H7), influenza A typing (pdH1N1, H3 and H3N2), influenza B (Yamagata and Victoria lineages), and RSV A and B by multiplex tandem RT-PCR. It also includes a human reference gene, NONO, and an artificial sequence, ‘spike’, to detect sample adequacy and inhibition, respectively. A positive control (AusDiagnostics respiratory virus control) and negative controls (DNase-free water; no template control) were included in each run.

Ten microlitres of eluted nucleic acid extract was added to assay tubes. These were run as per the manufacturer’s IFU on the High-Plex 24 System (AusDiagnostics), a commercial pathogen nucleic acid diagnostic platform based on multiplex tandem PCR (MT-PCR) technology [6]. The method involves a reverse transcriptase and pre-amplification step (15–18 cycles) to enrich all targets, a subsequent dilution step, and then a second PCR (one cycle at 95 °C for 10 min, 30 cycles at 95 °C for 10 s, 30 cycles at 60 °C for 20 s, 30 cycles at 72 °C for 10 s, and then 50 cycles at 75 °C for 5 s). In the second PCR step, specific gene targets are amplified using nested primers to increase specificity and sensitivity, and to reduce competition and non-specific amplification.

Automated curve interpretation was performed by the MT-Analyser software (AusDiagnostics). Amplification of the target gene within predetermined parameters was reported as ‘Present’. No amplification was reported as ‘Not detected’. When the cycling acceleration was slower than the pre-set parameters, it was reported as ‘Check’ and no concentration estimate was provided. This indicates that an operator’s involvement was required to investigate and interpret the result. The software also provided calculated Ct values, and viral concentration of the target molecule was calculated relative to the internal control SPIKE, expressed as arbitrary units or relative concentration [6].

### Limit of detection

A serial 1 : 10 dilution was performed on three positive samples with each sample dilution (1 : 10 to 1 : 100 000) extracted using both the QuickGene-810 and the EZ1 kit in parallel. Samples reflected a range of viral concentrations, and were chosen based on the original Ct values that were obtained from the MT-Analyser software when previously tested using the AusDiagnostics SARS-CoV-2, Influenza and RSV 8-well assay. Sample 1 was considered a low positive, sample 2 a mid-range positive and sample 3 a strong positive with high viral concentration.

### Statistical analysis

Agreement of the results between the two platforms was calculated: categorical overall percentage agreement (OPA), positive percentage agreement (PPA) and negative percentage agreement (NPA) were calculated with Microsoft Excel software. The effect of the different extraction platforms on Ct values was analysed by calculating the delta Ct (∆Ct) between platforms, its average and standard deviation (sd), with Microsoft Excel.

## Results

Of the 110 samples, there was a total of 27 positive and 83 negative samples using the EZ1 reference method. All of the results from QuickGene-810 extracts were concordant (see Table 1). The average difference in Ct values in positive samples differed between the QuickGene-810 and the EZ1 kit (see Appendix S1 for data by sample, available in the online version of this article). The average difference was 2.1±1.1 and 2.2±0.8 cycles for the *orf*1a and *orf*8 targets, respectively. The categorical performance characteristics demonstrate overall, percentage positive and percentage negative agreement were all 100 %.

**Table 1.** Comparison of the categorical detection of SARS-CoV-2 RNA from clinical samples extracted with the QuickGene-810 (Kurabo) vs. the EZ1 Advanced (Qiagen) and run on the SARS-CoV-2, Influenza and RSV 8-well assay (AusDiagnostics)

Extraction method		Qiagen EZ1 Advanced	
		Detected	Not Detected
Kurabo QuickGene-810	Detected	27	0
	Not detected	0	83

### Limit of detection

Comparison of the limit of detection (LOD) values (Tables 2–4) shows that the EZ1 Advanced kit has a lower LOD when compared to the QuickGene-810 kit, for all three samples, as determined by the AusDiagnostics results analysis software. This differs by one dilution factor for both targets (*orf*1a and *orf*8) in all three samples. Due to the volume of the positive samples being exhausted, the LOD was unable to be repeated with the same samples, and therefore reproducibility was not assessed.

**Table 2.** Comparison of the limit of detection (Ct values, as provided by the MT-Analyzer software; AusDiagnostics) of SARS-CoV-2 RNA from samples extracted with the QuickGene-810 (Kurabo) vs. the EZ1 Advanced (Qiagen) from samples with high, medium and low viral concentration, in dilution series run on the SARS-CoV-2, Influenza and RSV 8-well assay (AusDiagnostics)

Target	Sample concentration range	Extraction platform	Ct of neat sample	Ct of 1 : 10 dilution	Ct of 1 : 100 dilution	Ct of 1 : 1000 dilution	Ct of 1 : 10 000 dilution	Ct of 1 : 100 000 dilution
*orf*1a	High	QuickGene-810	20	23	29.27	31.08	–	–
		EZ1	17	21	25.71	29.09	32.45	–
–	Medium	QuickGene-810	32.05	–	–	–	–	–
		EZ1	27.93	32.18	33.21	–	–	–
	Low	QuickGene-810	–	–	–	–	–	–
		EZ1	31.64	–	–	–	–	–
*orf*8	High	QuickGene-810	18	21	27.06	27.97	33.11	–
		EZ1	15	19	23	26.39	29.9	32.19
	Medium	QuickGene-810	29.75	31.71				
		EZ1	25.82	29.19	32.88	–	–	–
	Low	QuickGene-810	31.95	–	–	–	–	–
		EZ1	29.73	31.54	–	–	–	–

**Table 3.** Comparison of the limit of detection (concentration, in relative concentration units, as provided by the MT-Analyzer software; AusDiagnostics) of SARS-CoV-2 RNA from samples extracted with the QuickGene-810 (Kurabo) vs. the EZ1 Advanced (Qiagen) from samples with high, medium and low viral concentration, in dilution series run on the SARS-CoV-2, Influenza and RSV 8-well assay (AusDiagnostics)

Target	Sample concentration range	Extraction platform	Concentration of neat sample	Concentration of 1 : 10 dilution	Concentration of 1 : 100 dilution	Concentration of 1 : 1000 dilution	Concentration of 1 : 10 000 dilution	Concentration of 1 : 100 000 dilution
*orf*1a	High	QuickGene-810	33 376	3805	84	29	–	–
		EZ1	172 806	18 349	537	69	8	–
	Medium	QuickGene-810	15	–	–	–	–	–
		EZ1	166	11	5	–	–	–
	Low	QuickGene-810	–	–	–	–	–	–
		EZ1	16	–	–	–	–	–
*orf*8	High	QuickGene-810	112 840	15 563	347	211	7	–
		EZ1	529 511	64 416	2928	392	39	11
	Medium	QuickGene-810	65	14	–	–	–	–
		EZ1	642	72	7	–	–	–
	Low	QuickGene-810	13	–	–	–	–	–
		EZ1	53	14	–	–	–	–

**Table 4.** Comparison of the limit of detection (qualitative) of SARS-CoV-2 RNA from samples extracted with the QuickGene-810 (Kurabo) vs. the EZ1 Advanced (Qiagen) from samples with high, medium and low viral concentration, in dilution series run on the SARS-CoV-2, Influenza and RSV 8-well assay (AusDiagnostics)

Target	Sample concentration range	Extraction platform	Result of neat sample	Result of 1 : 10 dilution	Result of 1 : 100 dilution	Result of 1 : 1000 dilution	Result of 1 : 10000 dilution	Result of 1 : 100000 dilution
*orf*1a	High	QuickGene-810	+	+	+	+	Check*	−
		EZ1	+	+	+	+	−	−
	Medium	QuickGene-810	+	Check*	−	−	−	−
		EZ1	+	+	+	−	−	−
	Low	QuickGene-810	−	−	−	−	−	−
		EZ1	+	−	−	−	−	−
*orf*8	High	QuickGene-810	+	+	+	+	+	−
		EZ1	+	+	+	+	+	+
	Medium	QuickGene-810	+	+	−	−	−	−
		EZ1	+	+	+	−	−	−
	Low	QuickGene-810	+	−	−	−	−	−
		EZ1	+	+	−	−	−	−

+, Sample result was positive by the assay’s software; −, negative result.

*As per the AusDiagnostics High-Plex IFU, a check is defined as a case when the cycling acceleration is less than the pre-set parameters and therefore no concentration estimate is provided. For this verification, a ‘check’ will be considered as positive as they are known positive samples.

## Discussion

Qualitative results from all clinical samples were concordant between the QuickGene-810 and the EZ1 kit extraction methods. Although the results matched, there was an average difference of approximately two cycles between the Ct values for both SARS-CoV-2 targets, suggesting that the EZ1 kit yields a higher concentration of nucleic acid extract. However, the different elution volumes (60 µl for EZ1 vs. 100 µl for Kurabo) could explain some of the difference in concentration. Therefore, it is recommended that in the case of a potential low positive or discrepancies between the two gene targets that re-extraction occur on another platform. In the dilution series, the samples that were discrepant between the QuickGene-810 and EZ1 kit had a very low viral concentration, of <20 relative units. Although this could reflect reduced sensitivity for infections with low viral load, patients with these low viral loads are much less likely to be infectious [7, 8]. With respect to ease of use, the QuickGene-810 kit displayed a greater person interaction time when compared to the EZ1 kit, as the QuickGene-810 kit is only semi-automated. Although the QuickGene-810 kit completes extraction faster (30 min), the increase in user operation and ability to only process eight samples per run is an important consideration if being used as a sole extraction platform. This is pertinent when high-throughput assays are required to meet the demand in testing, and with reduced staffing capacities. However, as a supportive platform for extraction it significantly increases the overall quantity of extracted samples.

There is no published information on the use of the QuickGene-810 platform in pathogen diagnostics, but the higher-throughput platform from the same manufacturer, the QuickGene-480, has been evaluated in a validation and field study of a mobile diagnostic laboratory in regional Western Australia for the COVID-19 pandemic response. It demonstrated a sensitivity of 100 % compared to the reference laboratory method and viral detection down to 2.80 copies μl^−1^ (2800 copies ml^–1^) [9]. There are a range of other well-established commercial extraction platforms used in pathogen diagnostics for the molecular detection of SARS-CoV-2 in respiratory samples, and all of the major commercial methods tested have been found to have good agreement, with recommendations that they could be interchanged [10–13]. The difficulties experienced worldwide in procuring laboratory equipment and reagents have necessitated diagnostic microbiology laboratories to explore other extraction and testing platforms on the market.

A limitation of this study was the relatively small sample size of 110 samples. At the time of conducting this comparison, the COVID-19 pandemic was in its early stages within Australia. Due to border closures and lockdowns, local case numbers were low, and obtaining positive samples at this time was challenging. The samples were not tested in replicates to assess reproducibility; this, along with the limited sample numbers, is a limitation and the results require reproducing in other settings.

For future research, the use of DTT and carrier rRNA could be considered as supplements to increase the yield of purified RNA. DTT acts to reduce the disulphide bonds of proteins, such as RNases, that degrade nucleic acid material [4]. Similarly, carrier RNA acts to increase the recovery of nucleic material through assisting RNA aggregation and precipitation [4]. The use of carrier RNA in the EZ1 kit procedure may explain some of the difference in Ct values and LOD seen between these extraction methods. In conclusion, the QuickGene-810 with the RNA Tissue Kit SII is a fit-for-purpose platform for SARS-CoV-2 RNA extraction from human respiratory samples. The platform performs comparatively with the EZ1 Advanced Extractor and is suitable for use with the SARS-CoV-2, Influenza and RSV 8-well assay, and by extrapolation would be suitable for use on other diagnostic platforms, including qPCR, after appropriate validation. In this study, there was agreement between qualitative assay results using these extraction platforms, despite higher Ct values and a higher LOD, which does infer the possibility of false-negative results for samples from patients with very late or very early infections with low viral loads, supported by discrepant values at low concentrations in our dilution series. However, these results support the introduction of the QuickGene-810 platform into diagnostic laboratories to support COVID-19 testing.
